# Menopause Literacy Gaps Across Individual, Healthcare and Societal Levels: A Qualitative Study of Women's Experiences and Healthcare Providers' Perspectives

**DOI:** 10.1111/hex.70938

**Published:** 2026-10-23

**Authors:** Najmeh Pourshahrokhi, Jamileh Farokhzadian, Myra S. Hunter, Saeedeh Azaraeen, Leila Ahmadian

**Affiliations:** ^1^ School of Allied Medical Sciences Jiroft University of Medical Sciences Jiroft Iran; ^2^ Department of Health Information Sciences, Faculty of Management and Medical Information Sciences Kerman University of Medical Sciences Kerman Iran; ^3^ Nursing Research Center Kerman University of Medical Sciences Kerman Iran; ^4^ Institute of Psychiatry, Psychology and Neuroscience King's College London London UK; ^5^ School of Medicine Jiroft University of Medical Sciences Jiroft Iran; ^6^ Fakher Mechatronic Research Center Kerman University of Medical Sciences Kerman Iran; ^7^ Research Management Centre INTI International University Putra Nilai Malaysia

**Keywords:** information needs, menopause, menopause literacy, patient education, qualitative research, women's health

## Abstract

**Background:**

Menopause is a major life transition that can affect women's physical, psychological, cognitive, sexual and social well‐being. Despite the availability of evidence‐based interventions, many women remain insufficiently informed and experience difficulties accessing reliable information and appropriate support.

**Objectives:**

To explore menopause‐related information needs and challenges from the perspectives of menopausal women and healthcare professionals and to identify individual, healthcare‐related and societal factors influencing menopause literacy.

**Design:**

A qualitative study informed by a constructivist paradigm was conducted using semi‐structured interviews and conventional qualitative content analysis.

**Methods:**

Twenty‐two peri‐ and postmenopausal women and 10 healthcare professionals were purposively recruited from healthcare settings in Southern Iran. Data were collected until data saturation was achieved, defined as the point at which no new codes or categories emerged from successive interviews. Interview transcripts were analysed using conventional qualitative content analysis following the approach of Graneheim and Lundman. Trustworthiness was enhanced through prolonged engagement, member checking, peer debriefing and established criteria for qualitative research.

**Results:**

Three interrelated themes were identified: understanding and accepting menopause, menopause management, and challenges of transition. Women described menopause as an unexpected and multidimensional transition affecting physical, psychological, cognitive, sexual, social and occupational well‐being. Both women and healthcare professionals identified substantial gaps in menopause literacy and emphasised the need for timely education, evidence‐based information and supportive healthcare. Symptom management strategies, lifestyle modification, family and peer support, and access to reliable information were perceived as important facilitators, whereas limited awareness, menopause‐related stigma, insufficient provider engagement and difficulty identifying trustworthy information were reported as major barriers.

**Conclusions:**

Menopause literacy is shaped by interacting individual, healthcare and societal factors. Strengthening evidence‐based menopause education, improving healthcare support and developing accessible, person‐centred information resources may enhance women's ability to navigate the menopausal transition and inform the design of future educational and digital health interventions.

## 1 Introduction

Menopause represents a natural biological transition marking the end of reproductive life [1]; however, women's experiences during this period extend beyond hormonal changes and are influenced by psychological, social and cultural contexts [2, 3, 4, 5]. Although the biological processes underlying menopause are relatively universal, perceptions, symptom interpretation, help‐seeking behaviours and coping strategies vary substantially across socio‐cultural environments [6, 7, 8]. Consequently, menopause should be understood as a biopsychosocial transition shaped by both individual experiences and the surrounding healthcare and social systems [2, 9, 10].

Adequate knowledge and understanding of menopause are essential for women to recognise symptoms, make informed decisions, seek appropriate care and adopt effective management strategies [11]. However, evidence from different countries indicates persistent gaps in menopause‐related knowledge among women [12, 13]. Many women enter the menopausal transition without adequate preparation and report uncertainty regarding symptom recognition, treatment options and lifestyle strategies [14, 15]. This lack of menopause‐related literacy may contribute to delayed help‐seeking, misconceptions about symptoms and reduced ability to actively participate in decisions regarding their health [12, 16].

Health literacy is increasingly recognised as a multidimensional concept extending beyond individual knowledge and encompassing the ability of individuals to access, understand, evaluate and use health information within supportive healthcare and social environments. Established frameworks, including Nutbeam's model and the integrated health literacy model proposed by Sørensen et al., emphasise that health literacy is influenced by interactions between personal capacities, healthcare systems and broader social determinants [17, 18]. Applying this perspective to menopause suggests that effective menopause literacy depends not only on women's awareness of symptoms and treatment options but also on healthcare providers' communication skills, availability of trustworthy information resources and societal attitudes towards menopause.

Despite growing recognition of menopause as an important women's health issue [9, 19], substantial gaps remain in the provision of menopause‐related education and support. Many women report limited access to evidence‐based information and frequently rely on family members, friends, social media and online resources, where information quality may be inconsistent [11, 12, 16]. Healthcare providers are expected to play a central role in supporting women during the menopausal transition; however, limited menopause‐specific training, time constraints, competing clinical priorities and the absence of standardised care pathways may hinder the delivery of comprehensive counselling [20, 21]. As a result, menopause care may focus primarily on symptom management rather than addressing women's broader informational, psychological and supportive needs.

Menopause experiences are also shaped by the socio‐cultural contexts in which women live. In many societies, menopause remains surrounded by stigma, silence and negative stereotypes that discourage open discussion and help‐seeking [22, 23]. Cultural beliefs about ageing, femininity, sexuality and women's social roles further influence how menopausal changes are interpreted and managed [23, 24]. These interacting social influences indicate that women's ability to access, understand and use menopause‐related information is determined not only by individual knowledge but also by the support provided by families, communities, healthcare systems and the wider social environment [17, 18].

Although menopause information needs have been explored in several countries [25, 26], evidence from culturally diverse and under‐represented settings remains limited. Several qualitative studies have explored Iranian women's experiences and perceptions of menopause, highlighting the influence of cultural beliefs, social expectations and personal experiences on the menopausal transition [23, 24, 26]. More recently, research has also examined health information‐seeking behaviour among Iranian menopausal women [27, 28]. However, existing evidence has largely focused on women's experiences or information‐seeking behaviours in isolation. Qualitative research integrating the perspectives of both menopausal women and healthcare providers to examine menopause‐related information needs within a broader health literacy perspective remains limited. Addressing this gap may contribute to a more comprehensive understanding of the individual, healthcare and socio‐cultural factors that shape women's access to, understanding of and use of menopause‐related information in Iran.

Understanding menopause‐related information needs requires exploring not only women's lived experiences but also the perspectives of healthcare professionals involved in menopause care. Therefore, this qualitative study aimed to explore menopause‐related information needs and challenges among menopausal women and healthcare professionals in Iran. Guided by established health literacy frameworks, the study explored individual, healthcare and socio‐cultural factors influencing women's ability to understand and manage menopause, providing an evidence‐informed foundation for future educational strategies, digital health interventions and policies to support person‐centred menopause care.

## 2 Methods

### 2.1 Study Design

This qualitative descriptive study employed a conventional qualitative content analysis approach to explore the information needs and experiences of menopausal women during the menopausal transition. The study was informed by a constructivist paradigm, which assumes that reality is socially constructed through individuals' lived experiences and interactions with their surrounding context [29]. This paradigm was considered appropriate because it enabled an in‐depth exploration of how women and healthcare professionals interpreted menopause‐related information needs within their socio‐cultural context.

The study included perspectives from both menopausal women and healthcare professionals to obtain a comprehensive understanding of menopausal information needs and challenges. Data collection and reporting were guided by the Consolidated Criteria for Reporting Qualitative Research (COREQ) checklist.

### 2.2 Settings and Participants

Participants were recruited from a variety of healthcare settings in Jiroft, Southern Iran, including primary healthcare centres, psychological counselling clinics, university‐affiliated general hospitals and private gynaecology clinics. A purposive sampling strategy with maximum variation was used to capture diverse experiences across age, educational level, marital status, occupation and menopausal stage.

Women were eligible if they were aged 45 years or older, were experiencing perimenopause or postmenopause, were able to communicate in Persian, and were willing to participate. Menopausal status was determined using participants' self‐reported menstrual history, consistent with the menstrual criteria described in the STRAW + 10 framework [3]. Women were classified as perimenopausal if they reported menstrual irregularity consistent with the menopausal transition and as postmenopausal if they reported at least 12 consecutive months of spontaneous amenorrhoea without another physiological or pathological cause. Because this qualitative study focused on women's experiences rather than clinical diagnosis, biochemical confirmation was not required. Women with premature ovarian insufficiency, early menopause (< 45 years) or surgically induced menopause were not included because these conditions have distinct clinical characteristics and information needs beyond the scope of the present study.

Healthcare professionals were eligible if they had at least 2 years of clinical experience in women's health or menopause‐related care and were directly involved in providing care for midlife women. All participating healthcare professionals were women and represented different disciplines, including gynaecology, reproductive health, midwifery, nursing, clinical psychology and social medicine.

Sampling continued until data saturation was achieved, defined as the stage at which no new codes, concepts or themes emerged from successive interviews. Saturation was monitored throughout data collection through regular discussions among the research team and was reached before recruitment ceased. Participant characteristics and eligibility criteria are presented in Table 1.

**Table 1:** Demographic characteristics of participants.

Menopausal women	Healthcare professional
– Age over 45 – Ability to communicate concepts and participate in interviews – No cognitive or behavioural disorders – No known hearing or speech problems – Being in peri‐menopause or post‐menopause – Experiencing or have experienced menopause symptoms – Willingness to participate in research	– Obstetricians and gynaecologists – Clinical psychologist – Reproductive health – Nursing – Midwifery – Mental health – Social medicine – Willingness to participate in research – At least 2 years of professional experience in women's health or menopause care – Employment in government organisations related to the community

### 2.3 Data Collection

Data were collected through individual, face‐to‐face, semi‐structured interviews conducted between July and December 2024. An interview guide was developed based on a literature review and discussions within the research team (Table 2). To enhance the suitability and comprehensiveness of the interview guide, three menopausal women with professional experience in midwifery and menopausal care reviewed and refined the guide, drawing on both their lived experiences and professional perspectives. Further details regarding their involvement in the study are provided in the ‘Patient and Public Involvement’ section.

**Table 2:** The interview guide.

Participants	Questions
Menopausal women	– How did menopause begin for you? – Describe the most important menopause symptoms you have experienced? – How much did you know about menopause and its changes before menopause began? – From what sources did you obtain information about menopause? – What information and training could you have needed to learn more about it to help you through this period? – How do you think information about menopause can be taught? – What challenges did you face along the way?
Healthcare professional	– What are your experiences with the menopausal transition in postmenopausal women? – In your opinion, what information and education do postmenopausal women need to go through menopause? – In your experience, what challenges do postmenopausal women face in going through menopause? – In your opinion, how can people's awareness about menopause be increased?
Probing questions	– Why? – How? – Explain more – Give an example

Prior to the main study, two pilot interviews were conducted to assess the clarity, flow and comprehensiveness of the interview guide. These pilot interviews led to minor revisions in the wording and sequencing of certain questions, as well as the addition of supplementary and probing questions. Participants in these pilot interviews were not included in the final study sample or the data analysis. Consequently, the final sample reported in this study consisted of 22 menopausal women and 10 healthcare professionals.

The interviews were conducted by the first author (N.P.). Before each interview, participants were informed about the researcher's academic background, the objectives of the study and the purpose of exploring menopause‐related information needs to improve menopause education and supportive care. At the beginning of each interview, demographic information, including age, educational level, occupation, marital status, parity, menstrual history and menopausal status, was collected. Open‐ended questions encouraged participants to describe their experiences, perceptions, challenges and information needs related to menopause. Follow‐up and probing questions were used to clarify meanings and facilitate deeper exploration of participants' experiences.

Interviews were conducted at a time and location chosen by the participants to ensure privacy and comfort. Each interview lasted approximately 30–60 min. With participants' written informed consent, all interviews were audio‐recorded and supplemented by field notes documenting contextual observations, non‐verbal communication and the interviewer's reflections.

Participants were informed that anonymised quotations from their interviews might be used in scientific publications, and explicit consent for the publication of anonymised verbatim quotations was obtained before data collection. Interviews included potentially sensitive topics related to menopause and were conducted in accordance with the study's ethical protocol.

Immediately after each interview, audio recordings were transcribed verbatim in Persian by the first author (N.P.). Audio recordings were retained only for the purpose of verifying transcription accuracy and ensuring analytical consistency during the study. They were securely stored on password‐protected computers accessible only to the research team. Personal identifiers were removed during transcription to maintain confidentiality. Quotations selected for publication were subsequently translated into English by a professional bilingual translator and independently reviewed by bilingual members of the research team to ensure conceptual equivalence and preserve participants' intended meanings. Any discrepancies were resolved through discussion until consensus was reached.

### 2.4 Data Analysis

Data were analysed using conventional qualitative content analysis following the approach described by Graneheim and Lundman [30]. This approach was selected because it allows systematic interpretation of participants' experiences through the identification of codes, categories and themes derived directly from the data.

Data collection and analysis were conducted concurrently. Analysis began immediately after each interview, allowing emerging insights to inform subsequent data collection. Verbatim Persian transcripts were read repeatedly by the first author (N.P.) to achieve immersion in the data and gain a comprehensive understanding of participants' experiences. Meaning units relevant to the study aim were identified, condensed while preserving their core meaning, and assigned initial codes.

Initial codes were compared according to similarities and differences and grouped into sub‐categories and broader categories. Through an iterative process of constant comparison across interviews, categories were progressively refined and abstracted into overarching themes that reflected the latent content of participants' experiences.

Coding and category development were performed independently by two researchers (N.P. and J.F.). The researchers met regularly to compare interpretations, discuss discrepancies and refine the coding framework, category structure and emerging themes. Whenever disagreements occurred, the original transcripts were revisited until consensus was achieved.

Data saturation was monitored throughout the analytical process. After each interview, newly generated codes were compared with those identified previously. Saturation was considered to have been achieved when no new codes, categories or concepts emerged from consecutive interviews and the existing categories provided a sufficiently comprehensive explanation of participants' experiences. Two additional interviews were conducted after saturation had been reached to confirm informational redundancy, and no new codes or categories were identified.

Because all interviews were conducted in Persian, coding and analysis were performed using the original Persian transcripts to preserve participants' meanings and cultural nuances. Only the quotations selected for publication were translated into English by a professional bilingual translator. The translated quotations were independently reviewed by bilingual members of the research team to ensure conceptual equivalence and consistency with the original Persian transcripts. Rather than producing literal translations, wording was refined where necessary to preserve the intended meaning of culturally specific or idiomatic expressions while maintaining readability in English.

Data management and organisation were facilitated using MAXQDA 2020 software (VERBI Software, Berlin, Germany). Triangulation was achieved through the integration of perspectives from menopausal women and healthcare professionals, together with investigator triangulation involving researchers from different disciplinary backgrounds.

### 2.5 Researcher Reflexivity

This study was conducted within a constructivist paradigm, acknowledging that qualitative findings are co‐constructed through interactions between researchers and participants. To enhance reflexivity, the research team included members from different disciplinary backgrounds, including medical informatics, reproductive health and qualitative health research, providing complementary perspectives throughout the study.

The interviews were conducted by the first author (N.P.), a female researcher with a PhD in medical informatics, experience in qualitative research, and a research focus on women's digital health and menopause. N.P. had no prior clinical or personal relationship with any participant before recruitment. The interviewer had a longstanding research interest in menopause and women's digital health, but maintained reflexive field notes and engaged in regular peer debriefing to minimise the influence of personal assumptions on data collection and interpretation. To enhance consistency while acknowledging the interactive nature of qualitative interviewing, a semi‐structured interview guide was used across interviews, and participants were encouraged to describe their experiences freely without leading questions or judgement.

Reflexive discussions were held throughout data collection and analysis to critically examine researchers' assumptions and interpretations. Coding decisions and emerging themes were regularly reviewed with the second qualitative researcher (J.F.), and discrepancies were resolved through discussion until consensus was reached. In addition, several members of the research team had lived experience of menopause. This experiential knowledge enriched the interpretation of participants' narratives while also prompting ongoing reflexive discussions to identify and critically examine potential assumptions throughout the analytical process. This combination of multidisciplinary expertise, collaborative analysis and continuous reflexive dialogue strengthened the credibility and confirmability of the findings.

### 2.6 Trustworthiness

The trustworthiness of the study was established according to the criteria proposed by Lincoln and Guba, including credibility, dependability, confirmability and transferability.

Credibility was enhanced through prolonged engagement with the data, purposive sampling with maximum variation and continued recruitment until data saturation was achieved. Member checking was conducted by sharing preliminary interpretations and summaries of the findings with a subset of participants to ensure that the emerging themes accurately reflected their experiences. In addition, regular peer debriefing sessions were held within the research team to discuss coding decisions, emerging categories and theme development.

Dependability was strengthened by maintaining a transparent and systematic analytical process. All stages of coding and theme development were documented, creating an audit trail that enabled the research process to be followed and evaluated. Coding was conducted independently by two researchers (N.P. and J.F.), and any disagreements were resolved through discussion until consensus was achieved.

Confirmability was promoted through investigator triangulation, reflexive discussions throughout the research process and documentation of analytical decisions. The multidisciplinary composition of the research team facilitated critical examination of interpretations and reduced the influence of individual researcher assumptions.

Transferability was supported by providing detailed descriptions of the study context, participant characteristics, sampling strategy, data collection procedures and analytical process, enabling readers to judge the applicability of the findings to other settings.

### 2.7 Patient and Public Involvement

Patient and public involvement was incorporated at two stages of the study [31]. Three menopausal women with professional experience in midwifery and menopause care were involved in reviewing and refining the interview guide before the main interviews. They provided feedback based on both their lived experience of menopause and their professional perspectives, which informed the relevance and comprehensiveness of the interview questions. Following data analysis, the same contributors also reviewed and confirmed the interpretation of the study findings. Their involvement, therefore, contributed to the refinement of the data collection process and the interpretation of the findings.

### 2.8 Ethical Considerations

Three menopausal women with professional experience in midwifery and menopause care were involved in the development and refinement of the interview guide. They reviewed the guide from both their lived experience of menopause and their professional perspectives and provided feedback on the relevance and comprehensiveness of the questions. They also participated in reviewing and confirming the interpretation of the study findings. Their involvement was incorporated into the study as part of the patient and public involvement process.

This study was approved by the Research Ethics Committee of Kerman University of Medical Sciences (Code: IR.KMU.REC.1403.087). In accordance with the committee's requirements, the research was conducted based on the principles of the Declaration of Helsinki and the guidelines of the Committee on Publication Ethics (COPE). Before participation, all participants received written and verbal information about the study objectives, interview procedures, voluntary nature of participation, confidentiality measures, and their right to withdraw from the study at any time without consequence. Written informed consent was obtained from all participants prior to data collection, including separate consent for audio recording and for the publication of anonymised quotations.

To protect participants' privacy, all interviews were anonymised during transcription by removing identifying information, and each participant was assigned a unique study code. Audio recordings and study documents were stored on password‐protected computers accessible only to the research team. In accordance with the information provided during the consent process, audio recordings will be permanently deleted following completion of the overall research project, whereas anonymised transcripts and analytical files will be retained securely in accordance with institutional research data management policies.

Because discussions about menopause could evoke emotional distress, a predefined distress procedure was followed during the interviews. If a participant appeared distressed or indicated discomfort, the interview was paused and the participant was given an opportunity to rest and decide whether to continue. The interviewer checked whether the participant wished to resume the interview, and the interview was terminated if the participant did not wish to continue. Participants could also decline to answer any question without consequence.

## 3 Results

### 3.1 Participant Characteristics

A total of 41 individuals (28 menopausal women and 13 healthcare professionals) were invited to participate. Of these, 32 participants completed the interviews, including 22 menopausal women (18 perimenopausal and 4 postmenopausal women) and 10 healthcare professionals, yielding an overall participation rate of approximately 78%. Reasons for non‐participation included lack of time, scheduling difficulties or declining participation.

Among the 22 menopausal women, 14 (63.6%) were married, and the mean age was 48.4 years. Eleven participants (50.0%) had an undergraduate degree, eight (36.4%) had a postgraduate degree and three (13.6%) had a diploma‐level education. Demographic characteristics of all participants are presented in Table S1.

### 3.2 Overview of Themes

The conventional content analysis generated three overarching themes reflecting women's information needs and experiences during the menopausal transition: (1) understanding and accepting menopause: experiences of transition and unmet informational needs, (2) menopause management: seeking information and support for symptom management, and (3) challenges of transitioning through menopause: individual, healthcare‐related and societal barriers. These themes comprised 7 categories and 20 sub‐categories. Findings are presented according to these themes, integrating the perspectives of menopausal women and healthcare providers.

The findings highlight that menopause was experienced not only as a biological transition but also as an informational and social process influenced by access to knowledge, support systems, healthcare interactions and broader socio‐cultural contexts. The themes, categories, sub‐categories and illustrative interview excerpts are presented in Tables 3, 4, 5. The results are presented according to the three themes, integrating women's lived experiences, healthcare providers' clinical perspectives and areas of convergence between the two groups.

**Table 3:** Interview excerpts supporting key factors related to the theme ‘Understanding and accepting menopause: experiences of transition and unmet informational needs’.

Theme	Category	Sub‐category	Perspective	Example participant quotes	Convergence
1. Understanding and accepting menopause	1.1. Unexpected recognition and lack of preparedness among women	1.1.1. Unexpected onset of menopause‐related changes	Women—lived experience	P4: ‘Even though I was a healthcare professional myself, I was shocked to encounter menopausal symptoms suddenly. I did many clinical tests; I didn't even suspect that I was approaching menopause’.	Both participant groups recognised that inadequate preparation before menopause contributed to uncertainty and emotional distress, highlighting the importance of timely education and supportive counselling to facilitate women's adaptation to the menopausal transition
		1.1.2. Feeling overwhelmed by unfamiliar menopausal changes	Women—lived experience	P8: ‘I was not prepared. It was like a tsunami hit my body and I was confused. I didn't know what was happening’. P1: ‘My symptoms started suddenly, with a pounding heart. For the next few days, I felt tired, and my body ached. I didn't have the patience for anyone. I lost control of myself quickly…. Nothing was in my control. I felt like things were happening in my body that I didn't know about’.	
		1.1.3. Need for timely education and supportive counselling	Providers—clinical perspective	P26 (Gynaecologist): ‘If women know in advance what symptoms to expect, it will prevent anxiety and worry caused by the sudden appearance of these symptoms and their repeated referrals to unrelated people and resources’. P28 (Psychologist): ‘Many doctors may simply not talk to their patients about the natural changes that occur during menopause. This can make women feel like the problems they are experiencing are worrisome and unnatural’.	
	1.2. Multidimensional impact of menopausal transition on women's lives	1.2.1. Physical and functional consequences	Providers—clinical perspective	P25 (Gynaecologist): ‘Night sweats in women can be so annoying that they completely disrupt a person's sleep’.	Both menopausal women and healthcare professionals described menopause as a multidimensional transition with interconnected physical, psychological, cognitive, sexual and social consequences that substantially affect women's daily functioning and quality of life
			Women—lived experience	P2: ‘My leg and body pains made me feel like I was getting too old. I used to walk a lot; now I get tired easily, and I can't even do the usual housework’.	
		1.2.2. Psychological and emotional consequences	Women—lived experience	P16: ‘My house has always been busy … but now I don't feel like having anyone over. I prefer to be alone’.	
			Providers—clinical perspective	P28 (Psychologist): ‘Hormonal changes can affect a woman's mood and make depression and anxiety worse’.	
		1.2.3. Sexual health and intimate relationship challenges	Women—lived experience	P10: ‘The pain during sex has made me never want to have sex with my wife. If I have sex, the pain is accompanied by a feeling of disgust, and this has made my wife avoid me’.	
			Providers—clinical perspective	P31 (Midwife): ‘Postmenopausal women may feel like they have lost their libido due to hormonal changes and may also experience vaginal dryness, but there are solutions to manage these symptoms’.	
		1.2.4. Cognitive difficulties and occupational impact	Women—lived experience	P7: ‘As a university professor, I couldn't concentrate in class and would forget my sentences. I felt like I wasn't capable of teaching’.	
			Women—lived experience	P12: ‘I couldn't concentrate on my work the next day. I was exhausted…. I felt like I couldn't do my job anymore. I was worried I would lose my job’.	

**Table 4:** Interview excerpts supporting key factors related to the theme ‘Menopause management: seeking information and support for symptom management’.

Theme	Category	Sub‐category	Perspective	Example participant quotes	Convergence
2. Menopause management	2.1. Symptom management strategies	2.1.1. Treatment strategies and uncertainty	Women—lived experience	P9: ‘I have been looking for a way to manage my hot flashes for a long time. menopausal hormone therapy (MHT) was a good option for me, but I was worried about the side effects, and I was afraid of getting cancer’.	Both groups emphasised that menopause management requires personalised information and guidance rather than a single standardised approach
			Providers—clinical perspective	P26 (Gynaecologist): ‘Every menopausal woman should keep in mind that there is no one‐size‐fits‐all solution. We need to look at each person's medical history and symptoms to identify the best treatment option’.	
		2.1.2. Lifestyle modification and self‐management	Women—lived experience	P19: ‘Regular walking saved me. Not only did it make my body stronger, but I also think and dream less; I deal with my children in a better mood’.	Both groups recognised lifestyle changes as practical and accessible strategies for improving symptoms and overall well‐being
			Providers—clinical perspective	P23 (Nurse): ‘If women eat a diet that includes whole grains, higher protein, high vegetables and no sugar, they can manage their menopause well’.	
			Women—lived experience	P7: ‘I didn't eat heavy meals at night. I tried not to eat fried food. I had a light meal around 8 o'clock’.	
	2.2. Information sources and knowledge acquisition	2.2.1. Family experiences as early menopause knowledge sources and emotional support	Women—lived experience	P14: ‘Everything I learned about menopause came from my mother's experiences. Menopause was a lot of changes for my mother. Her experience of mood swings, sudden fatigue and hot flashes made me look at this issue more carefully’. P15: ‘After consulting with my doctor, the support of my family really helped me when I talked to my husband and son about the changes in my body’.	Women described family members as an important early source of menopause‐related knowledge and emotional support, highlighting the role of intergenerational experiences in shaping menopause awareness
		2.2.2. Peer interaction and shared experiences as coping resources	Women—lived experience Providers—clinical perspective	P22: ‘Participating in yoga has truly changed my life. I have made so many friends…. It has given me a sense of peace to see that other women are going through the same things I am going through’. P30 (Mental health experts): ‘It is important for women to connect during this period and feel that they are not alone in this stage of life, and it helps maintain their peace of mind’.	Both women and healthcare professionals recognised peer interaction and shared experiences as valuable sources of emotional reassurance and practical coping during the menopausal transition
		2.2.3. Digital media as an accessible but potentially unreliable information source	Women—lived experience	P21: ‘After I found out I was going through menopause; I was always looking for accurate information about my physical changes. The internet has helped me find articles and opinions from doctors and experts easily, which has made me feel more confident’.	Both women and healthcare professionals acknowledged that digital media provides convenient access to menopause information while emphasising the importance of using trustworthy evidence‐based sources to avoid confusion and misinformation
			Providers—clinical perspective Convergence	P29 (Psychologist): ‘Social media can be a helpful resource, but information needs to be verified from reliable sources to avoid confusing women’.	

*Note:* Convergence—Both participant groups highlighted that menopause knowledge is acquired through multiple complementary sources, including family experiences, peer interactions and digital media, while emphasising the importance of accessing trustworthy evidence‐based information.

**Table 5:** Interview excerpts supporting key factors related to the theme ‘Challenges of transitioning through menopause: individual, healthcare‐related and societal barriers’.

Theme	Category	Sub‐category	Perspective	Example participant quotes	Convergence
3. Challenges of transitioning through menopause	3.1. Individual‐level barriers: Limited knowledge and preparedness	3.1.1. Lack of prior menopause education and awareness	Women—lived experience	P11: ‘I really don't know much about it. There is no education or talk about menopause in school, college, on TV or in the media. It's as if it doesn't matter how half of society lives’.	Both participant groups recognised that limited menopause literacy, inadequate preparation before symptom onset and difficulty identifying reliable information sources reduced women's confidence in recognising and managing menopausal changes
			Women—lived experience	P5: ‘We didn't know anything about menopause except what we learned firsthand or indirectly from people who had experienced it’.	
			Providers—clinical perspective	P23 (Nurse): ‘Menopause should be taught to boys and girls in school at a young age. If boys, who are future men, know more, they can better support their mothers and wives in the future’.	
			Providers—clinical perspective	P27 (Social medicine): ‘I believe that education about menopause and preparation for it should begin years before women experience menopausal symptoms, rather than only after those symptoms appear’.	
		3.1.2. Difficulty identifying reliable information sources	Women—lived experience	P18: ‘When I approached menopause, I didn't know anything; everything was vague. I didn't know where or who to ask for help. Everyone was saying, “Just deal with it, that's what menopause is all about!”’	
			Women—lived experience	P16: ‘When I was looking for the right information about menopause symptoms, I didn't know how to find the right information or which source to trust’.	
	3.2. Healthcare provider and system‐level barriers: Gaps in professional support	3.2.1. Limited menopause‐related knowledge and counselling among healthcare providers	Women—lived experience	P12: ‘My symptoms suddenly started with palpitations, anxiety, and depression. I went to many doctors and had many tests…. No one told me that these were menopause symptoms’.	Both women and healthcare professionals identified insufficient menopause‐related counselling, limited provider knowledge and time constraints during clinical consultations as important barriers to effective menopause care and informed decision‐making
			Women—lived experience	P13: ‘When I went to my doctor, I felt like he didn't know anything about menopause and its challenges. I really needed my doctor to know more about my mood swings and sleep problems’.	
		3.2.2. Time constraints and limited opportunities for comprehensive consultation	Providers—clinical perspective	P25 (Gynaecologist): ‘Limited time prevents me from properly addressing all of my patients' problems. I feel that I do not have enough time to talk about menopause and this prevents me from providing a comprehensive approach’.	
			Women—lived experience	P16: ‘The lack of time not only affects the quality of the consultation, but also makes us feel that our problems are not understood’.	
	3.3. Societal, cultural and information environment barriers: Stigma, silence, insufficient support and digital misinformation	3.3.1. Menopause as a taboo topic	Women—lived experience	P18: ‘Sometimes I feel like I have to keep quiet about my problems because I don't want to expose myself to judgment and ridicule’.	Both participant groups highlighted that socio‐cultural stigma, limited public awareness, inadequate social support and difficulties accessing trustworthy digital health information collectively hinder women's ability to seek information, discuss menopause openly and navigate the menopausal transition
			Women—lived experience	P7: ‘My mother had never talked to me about menopause, and I was completely unaware of it’.	
		3.3.2. Gender stereotypes and workplace challenges	Women—lived experience	P15: ‘At work, when I had symptoms, I couldn't talk to my manager about my problems because he was a man and he couldn't understand me’.	
			Women—lived experience	P13: ‘I always thought that if I tried to talk about my problems, I would be seen as a weak woman and lose my job position’.	
		3.3.3. Lack of social awareness and support	Providers—clinical perspective	P30 (Mental health experts): ‘If society had a better understanding of menopause and considered it a normal part of life, menopausal women would be more comfortable talking about it’.	
			Women—lived experience	P21: ‘I felt completely alone in a society where it's ugly to talk about menopause…. Sometimes I wondered if anyone really understood what was going on with me’.	
		3.3.4. Inadequate and unreliable digital information environment	Women—lived experience	P2: ‘When I went online, I felt more confused than I learned’.	
			Providers—clinical perspective	P28 (Psychologist): ‘Women going through menopause are looking for effective ways to manage their symptoms, but the lack of information available online can lead to confusion and anxiety’.	
			Women—lived experience	P14: ‘My friends and I were always looking for reliable sources about menopause, but we were confused by not knowing where to find this information’.	
			Providers—clinical perspective	P24 (Psychologist): ‘The lack of reliable sources leads women to turn to misinformation. This not only confuses them, but also increases their stress and anxiety’.	

### 3.3 Theme 1: Understanding and Accepting Menopause: Experiences of Transition and Unmet Informational Needs

Participants described menopause as a life transition that was frequently unexpected and poorly understood. Women consistently reported that the onset of menopausal symptoms occurred without adequate prior knowledge, leaving them confused, anxious and unprepared to recognise the changes they were experiencing. Many interpreted early symptoms as signs of illness rather than menopause, resulting in repeated medical consultations and considerable emotional distress. Several participants reflected that receiving education before the onset of menopause would have reduced uncertainty and facilitated earlier recognition of menopausal changes.

Beyond the initial recognition of menopause, women described the substantial impact of menopausal symptoms on multiple aspects of daily life. Physical symptoms, including vasomotor symptoms, musculoskeletal pain, fatigue and sleep disturbances, often disrupted routine activities and reduced functional capacity. Participants also reported significant psychological consequences, including anxiety, irritability, depressed mood, emotional instability and feelings of losing control. Sexual health concerns, such as decreased libido, vaginal dryness and dyspareunia, negatively affected intimate relationships, while cognitive complaints, including forgetfulness, impaired concentration and brain fog, interfered with occupational performance and self‐confidence.

Healthcare professionals largely confirmed these experiences. They emphasised that many women enter menopause without adequate knowledge of expected physiological and psychological changes and highlighted the importance of anticipatory counselling before symptom onset. Providers further acknowledged the broad physical, psychological, sexual and cognitive consequences of menopause and stressed that early education could reduce unnecessary visits to healthcare services, alleviate anxiety and improve women's ability to adapt to the menopausal transition.

Overall, findings from both participant groups converged in demonstrating that timely, evidence‐based menopause education is fundamental to promoting women's understanding and acceptance of menopause. Early recognition of menopausal changes was viewed as the first step towards effective self‐management and a more positive transition experience (Table 3).

### 3.4 Theme 2: Menopause Management: Seeking Information and Support for Symptom Management

Women described menopause management as an active process that extended beyond symptom control to include informed decision‐making, lifestyle modification, social support and continuous information seeking. Rather than relying on a single strategy, participants reported combining pharmacological and non‐pharmacological approaches according to their individual symptoms, preferences and personal beliefs. While some women considered hormone therapy an effective option for relieving menopausal symptoms, concerns about potential adverse effects often led them to seek alternative or complementary approaches. Healthcare professionals similarly emphasised that menopause management should be individualised and based on each woman's symptoms, medical history and treatment preferences.

Lifestyle modification emerged as another central strategy for symptom management. Women described adopting regular physical activity, healthier dietary habits and improved daily routines to reduce vasomotor symptoms, improve sleep quality, maintain emotional well‐being and enhance their overall quality of life. Healthcare providers supported these experiences by recommending healthy nutrition and regular exercise as essential components of menopause care.

Participants also highlighted the importance of social support throughout the menopausal transition. Family members, particularly spouses, sisters and mothers, provided emotional reassurance and practical guidance, while friends and peer networks helped normalise menopausal experiences through shared understanding. Participation in community‐based activities, such as yoga groups, further reduced feelings of isolation and promoted psychological well‐being. Healthcare professionals likewise emphasised that supportive social relationships enable women to exchange experiences, strengthen coping strategies and navigate menopause more confidently.

Women obtained menopause‐related information from a variety of interpersonal and digital sources, including family members, friends, healthcare professionals, the internet and social media. Although online resources increased access to menopause information, participants expressed concerns regarding the credibility and consistency of available information. Healthcare professionals similarly acknowledged the growing role of digital media while emphasising that educational resources should be evidence‐based and scientifically reliable.

Overall, women and healthcare professionals shared the view that effective menopause management requires a comprehensive and individualised approach that integrates appropriate medical care, healthy lifestyle behaviours, supportive interpersonal relationships and access to trustworthy health information (Table 4).

### 3.5 Theme 3: Challenges of Transitioning Through Menopause: Individual, Healthcare‐Related and Societal Barriers

Women and healthcare professionals consistently described multiple barriers that limited women's ability to obtain appropriate menopause information and support. These barriers operated at the individual; healthcare provider and system‐level; and societal, cultural and information environment barriers, illustrating that the challenges of menopause extended beyond personal symptom management.

### 3.6 Individual‐Level Barriers

Women described entering menopause with limited knowledge of menopausal changes, making it difficult to recognise symptoms and seek appropriate support. Many participants initially attributed psychological and physical symptoms to unrelated illnesses rather than menopause. Uncertainty about where to obtain reliable information and exposure to conflicting online resources further increased confusion and anxiety. Participants frequently reported feeling overwhelmed by contradictory advice and expressed difficulty distinguishing trustworthy sources from misinformation.

### 3.7 Healthcare Provider and System‐Level Barriers

Women also encountered important barriers within healthcare services. Participants described repeated consultations in which menopausal symptoms were overlooked or attributed to unrelated conditions, delaying appropriate management. Some women felt that healthcare providers lacked sufficient knowledge regarding menopause, while others emphasised that short consultation times prevented meaningful discussion of their concerns. Healthcare professionals acknowledged these challenges, explaining that heavy workloads and limited consultation time restricted opportunities to provide comprehensive menopause counselling. They emphasised the need for dedicated menopause education and specialised services capable of addressing women's informational and emotional needs.

### 3.8 Societal, Cultural and Information Environment Barriers

Beyond individual and healthcare‐related factors, both groups identified broader social and cultural barriers that hindered successful menopause transition. Participants described menopause as a stigmatised topic rarely discussed within families, workplaces or the wider community, leading many women to conceal their symptoms because of embarrassment or fear of negative judgement. Workplace environments were often perceived as lacking understanding and support, while gender stereotypes contributed to women's concerns about appearing weak or less competent. Healthcare professionals further emphasised that improving menopause literacy should extend beyond women themselves and include men, family members, employers, schools and the wider community. Both groups agreed that reducing stigma and expanding access to reliable, evidence‐based educational resources are essential to creating a more supportive environment for women during the menopausal transition (Table 5).

## 4 Discussion

### 4.1 Principal Findings

This study explored the information needs and experiences of menopausal women from the perspectives of both women and healthcare professionals. Rather than identifying isolated educational needs, the findings revealed a progressive pathway through which women experience the menopausal transition. Participants consistently described menopause as a process that begins with uncertainty and limited recognition of early changes, continues through attempts to understand and manage a wide range of physical, psychological, cognitive, sexual and social consequences, and is further complicated by barriers operating at individual, healthcare and societal levels. Across all three themes, women and healthcare professionals expressed remarkably consistent views regarding the importance of timely, evidence‐based and accessible menopause education.

The first theme highlighted that many women entered the menopausal transition without sufficient prior knowledge, resulting in confusion, anxiety and delayed recognition of menopause‐related changes. Women frequently interpreted early symptoms as signs of illness rather than a normal life transition and often sought repeated medical consultations before menopause was considered as a possible explanation. Healthcare professionals similarly acknowledged that anticipatory counselling is rarely provided before symptom onset and emphasised that earlier education could reduce unnecessary anxiety, improve symptom recognition and facilitate a smoother transition. These findings suggest that preparation for menopause should begin before symptoms emerge rather than after women actively seek healthcare.

The second theme demonstrated that information needs extend well beyond symptom recognition. Women sought practical guidance for managing menopausal symptoms through both medical and non‐pharmacological approaches, while also relying on multiple sources of information, including family members, peers, digital media and healthcare professionals. Participants recognised the value of these resources but also questioned their credibility and consistency. Healthcare professionals similarly emphasised that management strategies should be individualised and supported by reliable evidence. Together, these findings indicate that women require not only information about menopause itself but also support in evaluating information quality and selecting appropriate management options according to their individual circumstances.

The third theme revealed that informational needs cannot be understood independently of the broader context in which women experience menopause. Participants described multiple barriers that limited access to appropriate knowledge and support, including insufficient education, limited consultation time, inadequate professional knowledge, stigma surrounding menopause, workplace misunderstanding, gender‐related stereotypes and difficulties identifying trustworthy information. Importantly, these barriers operated across several interacting levels, encompassing women themselves, healthcare providers, healthcare systems, workplaces, families and the wider community. The convergence of women's lived experiences with healthcare professionals' observations suggests that improving menopause literacy requires coordinated interventions extending beyond clinical consultations alone.

Taken together, these findings support a broader conceptual understanding of menopause literacy as a multidimensional construct. Women's information needs are not confined to acquiring factual knowledge about menopausal symptoms; rather, they involve developing the knowledge, skills, confidence and supportive environments necessary to recognise menopausal changes, make informed management decisions, navigate available information resources and seek appropriate support throughout the menopausal transition. This integrated perspective provides a foundation for developing comprehensive educational and digital interventions that address menopause across individual, healthcare and societal contexts.

### 4.2 Comparison With Previous Studies

The findings of this study are broadly consistent with previous qualitative research showing that women often enter the menopausal transition with limited knowledge and insufficient preparation [32]. However, the present study extends existing evidence by demonstrating that women's informational needs are not confined to learning about menopausal symptoms. Rather, these needs evolve throughout the menopausal transition and are shaped by interactions between personal experiences, healthcare encounters and broader social contexts.

Our first theme highlighted that many women experienced menopause as an unexpected and confusing life transition. Participants frequently interpreted early physical and psychological symptoms as signs of illness rather than manifestations of menopause, resulting in anxiety, repeated healthcare consultations and delayed recognition of menopausal changes. Importantly, participants' descriptions of menopause as occurring ‘suddenly’ should not be interpreted as indicating an abrupt biological onset. According to the STRAW + 10 framework, the menopausal transition is typically a gradual process that unfolds over several years. The present findings instead suggest that many women did not recognise early menopausal changes until symptoms accumulated and became sufficiently disruptive to prompt healthcare seeking. Consequently, menopause was retrospectively perceived as an unexpected event rather than a gradual transition. This interpretation reinforces the central finding of the present study that limited menopause literacy contributes to delayed symptom recognition and highlights the importance of anticipatory education before symptom onset. Similar findings have been reported in qualitative studies from different cultural settings, where women described feeling unprepared for menopause and expressed a strong need for anticipatory education before symptom onset [33, 34]. Recent qualitative reviews have also concluded that inadequate menopause literacy contributes to uncertainty, emotional distress and delayed help‐seeking during the menopausal transition [35].

Our findings also support previous evidence that menopause education should extend beyond improving symptom recognition [36]. Women in the present study consistently sought practical information on treatment options, lifestyle modification and self‐management strategies, while simultaneously attempting to judge the credibility of information obtained from family members, peers, healthcare professionals and digital resources. An important finding was that some women expressed concerns about menopausal hormone therapy based on fears of cancer. Although these concerns reflect participants' lived experiences and should be acknowledged, they also illustrate how outdated or inaccurate information may influence decision‐making. Misconceptions surrounding menopausal hormone therapy have persisted since the initial publication of the Women's Health Initiative (WHI) study, despite substantial re‐evaluation of the evidence. Contemporary international guidelines emphasise that, for women who are younger than 60 years or within 10 years of menopause onset and who have no contraindications, menopausal hormone therapy is an effective and appropriate treatment for vasomotor symptoms when individualised risk–benefit assessment and shared decision‐making are undertaken [9, 37, 38, 39]. These findings reinforce the importance of evidence‐based menopause education to address persistent misinformation and support informed treatment decisions.

Healthcare professionals similarly emphasised the importance of individualised, evidence‐based counselling rather than a single management approach. These observations are consistent with previous qualitative research demonstrating that women value reliable and personalised information and often combine professional advice with experiential knowledge obtained through social networks and online resources [33]. At the same time, previous studies have shown that inconsistent or commercially driven online information may increase uncertainty and make informed decision‐making more difficult [40].

Another important finding concerns the role of healthcare professionals. The inclusion of both menopausal women and healthcare professionals also provided opportunities for triangulation of perspectives. Although both groups consistently identified inadequate menopause education and limited access to reliable information as major barriers, their interpretations differed in important ways. Women primarily described these barriers through their lived experiences of uncertainty, delayed symptom recognition and emotional distress, whereas healthcare professionals emphasised structural constraints such as limited consultation time, insufficient menopause‐specific training and the absence of standardised educational resources. The convergence of these perspectives strengthens confidence in the identified information needs, while their differences highlight that improving menopause literacy requires interventions targeting both women's educational needs and healthcare system capacity.

Although participants viewed healthcare providers as the most trusted source of information, many women described delayed diagnosis, inadequate counselling and consultations focused primarily on pharmacological treatment rather than comprehensive menopause care. Healthcare professionals participating in our study acknowledged similar challenges, particularly limited consultation time and variable professional knowledge. These findings closely align with recent qualitative studies reporting that many clinicians lack confidence in recognising the full spectrum of menopausal symptoms and often experience organisational constraints that limit opportunities for patient‐centred counselling [41, 42]. Such barriers may contribute to repeated healthcare utilisation and unmet informational needs among menopausal women [43].

The third theme demonstrated that menopause literacy is strongly influenced by social and cultural environments. Participants described stigma, embarrassment, gender stereotypes, inadequate workplace support and limited family understanding as major barriers to discussing menopause and seeking help. Similar findings have been reported internationally, where fear of negative judgement frequently discourages women from disclosing menopausal symptoms, particularly within professional settings [44]. Recent qualitative syntheses further suggest that organisational culture and workplace awareness substantially influence women's experiences during the menopausal transition and should be considered integral components of menopause support rather than peripheral issues [45, 46].

An additional contribution of this study concerns digital information seeking. Participants frequently relied on the internet and social media because these resources were readily accessible when professional advice was unavailable or insufficient. Nevertheless, women described considerable difficulty distinguishing trustworthy information from conflicting or inaccurate online content. In contrast, healthcare professionals emphasised the limited availability of reliable, evidence‐based educational resources that could be confidently recommended during routine clinical consultations. Together, these complementary perspectives suggest that improving menopause literacy requires not only increasing access to digital information but also ensuring the availability of trustworthy, evidence‐based resources that support both women and healthcare providers. Similar concerns have been highlighted in recent qualitative research, which emphasises that improving menopause literacy requires not only increasing information availability but also strengthening women's ability to evaluate information quality and navigate increasingly complex digital health environments [35, 45, 46].

Taken together, these findings suggest that understanding menopause through a health literacy perspective may provide a broader conceptual lens for interpreting women's information needs across individual, healthcare and societal contexts. By incorporating the perspectives of both menopausal women and healthcare professionals, this study demonstrates that menopause literacy develops through continuous interactions between individual knowledge, healthcare systems and socio‐cultural environments. This integrated perspective offers a broader understanding of women's informational needs and provides a stronger theoretical foundation for designing comprehensive educational programmes and evidence‐based digital health interventions that address menopause across multiple levels simultaneously.

### 4.3 Implications for Practice and Policy

The findings of this study have several implications for clinical practice, health policy and the development of future menopause education programmes. First, menopause education should shift from a reactive model, in which information is provided only after women seek help for symptoms, to a proactive and anticipatory approach. Participants consistently reported that the absence of timely information contributed to uncertainty, delayed symptom recognition, unnecessary healthcare utilisation and emotional distress. Integrating menopause education into routine primary healthcare services before the onset of menopause could facilitate earlier recognition of menopausal changes and support informed decision‐making throughout the transition. This recommendation is consistent with current international guidance, including the NICE guideline on menopause identification and management (NG23) and the 2024 Lancet menopause series, which emphasise timely education and informed decision‐making across the menopausal transition [9, 37, 38].

Second, the findings highlight the need to strengthen menopause‐related competencies among healthcare professionals, particularly those working in primary care. Women frequently described consultations in which menopausal symptoms were overlooked, misinterpreted or addressed only through symptom‐based treatment [47]. Continuing professional education and the implementation of evidence‐based clinical guidance could improve healthcare providers' confidence in recognising menopausal symptoms, delivering person‐centred counselling and discussing both pharmacological and non‐pharmacological management options [48]. Such improvements may enhance communication between women and healthcare professionals while reducing unmet informational needs. These findings align with recommendations from the 2022 Position Statement of the Menopause Society [39], NAMS 2022 hormone therapy position statement [49], the International Menopause Society White Paper [50] and the NICE guideline [37], all of which advocate individualised assessment, shared decision‐making and evidence‐based counselling tailored to women's preferences and clinical circumstances [48].

Third, our findings suggest that menopause literacy should be considered a shared societal responsibility rather than solely an individual concern. Participants emphasised the importance of increasing awareness among partners, family members, employers, educators and the wider community. Public health initiatives that normalise conversations about menopause and reduce menopause‐related stigma may encourage women to seek support earlier and promote more supportive family, workplace and community environments. Recent international guidance similarly recognises that improving menopause care requires coordinated action extending beyond clinical services to include workplaces, communities and public health systems [51, 52].

Finally, the findings support the development of comprehensive digital health interventions that provide accessible, evidence‐based and personalised menopause education [53, 54]. Although many women actively sought information through digital platforms, they often encountered inconsistent or unreliable content and experienced difficulty identifying trustworthy sources. Digital interventions designed according to women's informational needs and developed in collaboration with healthcare professionals may help address these gaps by providing reliable educational resources, personalised self‐management support, symptom monitoring and timely access to evidence‐based recommendations. Such interventions have the potential to complement routine healthcare services while improving access to menopause education, particularly for women who experience geographical, organisational or time‐related barriers to care.

Overall, these findings support the adoption of a multi‐level approach to menopause education that combines patient education, professional training, health system strengthening, community awareness and evidence‐based digital solutions. Addressing these interconnected levels simultaneously is likely to improve menopause literacy, facilitate informed self‐management and promote more equitable access to menopause support throughout the menopausal transition. Collectively, these recommendations are consistent with contemporary international menopause guidance and reinforce the importance of integrating evidence‐based, person‐centred menopause care into routine healthcare and public health practice [9, 37, 39, 48, 55].

### 4.4 Strengths and Limitations

This study has several important strengths. First, it explored menopause‐related information needs from the perspectives of both menopausal women and healthcare professionals, providing a more comprehensive understanding than studies focusing on a single stakeholder group. Triangulating these complementary perspectives allowed convergence and divergence in experiences and expectations to be identified, thereby strengthening the interpretability of the findings.

Second, participants represented diverse educational backgrounds, professional disciplines and menopausal experiences. Including healthcare professionals from multiple fields—including obstetrics and gynaecology, midwifery, nursing, psychology, reproductive health, mental health and social medicine—enabled the study to capture a broad range of clinical and educational perspectives. This diversity enriched the analysis and supported the development of a more comprehensive conceptual understanding of menopause‐related information needs.

Third, rather than producing a descriptive list of educational topics, the study generated an integrated conceptual framework that organised women's information needs across three interconnected domains: understanding and accepting menopause, managing the menopausal transition, and addressing barriers to obtaining information and support. This framework provides a practical foundation for developing evidence‐based educational programmes, digital health interventions and future implementation research.

Several limitations should be acknowledged. First, this study was conducted in a single geographical region in Southern Iran. Participants were recruited from primary healthcare centres, gynaecology clinics and psychological counselling clinics, and most women had university‐level education. Although purposive sampling with maximum variation was used, voluntary participation may have resulted in under‐representation of women with lower educational attainment, limited engagement with healthcare services or less willingness to discuss menopause. Consequently, the findings should be interpreted within the study context, and their transferability to other cultural, educational and healthcare settings should be considered with caution.

Second, the scope of this study was intentionally limited to women experiencing natural perimenopause and postmenopause. Women with premature ovarian insufficiency, early menopause (< 45 years) and surgically induced menopause were excluded because these populations have distinct clinical characteristics, treatment pathways and information needs and may require different educational and support strategies. Therefore, the transferability of the present findings to these populations is limited.

Third, although all participating healthcare professionals were female, the perspectives of male healthcare providers were not represented in this study. Participants highlighted the importance of improving menopause awareness among partners, employers, colleagues and family members; these perspectives were derived exclusively from interviews with menopausal women and healthcare professionals. Other stakeholder groups were not interviewed directly; therefore, their experiences, informational needs and perceptions were not explored. Future research should include these groups to provide a more comprehensive understanding of the broader social context influencing menopause literacy and support.

Finally, this qualitative study was designed to explore information needs and experiences rather than evaluate the effectiveness of specific educational or digital interventions. Accordingly, the findings should be interpreted as providing an empirical foundation for intervention development rather than evidence of intervention effectiveness. Future research should build on these findings by developing and evaluating theory‐informed educational programmes and digital health interventions that address menopause literacy across individual, healthcare and societal levels.

### 4.5 Future Research

The present findings provide a foundation for future research aimed at improving menopause education and support across multiple levels. Future studies should develop and evaluate theory‐informed educational interventions that address the multidimensional information needs identified in this study. Such interventions should extend beyond symptom education to include support for self‐management, communication with healthcare professionals, evaluation of health information and navigation of available healthcare resources.

Future research should also examine the effectiveness of digital health interventions, including mobile applications, web‐based educational programmes and decision‐support tools, in improving menopause literacy, self‐management behaviours and health outcomes. Given the barriers identified by participants, digital interventions should be co‐designed with end users and healthcare professionals to ensure that they provide accessible, personalised, culturally appropriate and evidence‐based information.

Future research should include women from diverse educational, socio‐economic and cultural backgrounds, as well as those with varying levels of engagement with healthcare services, to provide a more comprehensive understanding of menopause literacy across different population groups. Multi‐site qualitative and quantitative studies conducted across diverse healthcare and socio‐cultural settings are also needed to examine the transferability of the present findings and to explore how contextual factors shape menopause‐related information needs. Comparative research across countries and healthcare systems may further inform the development of contextually adaptable menopause education strategies while acknowledging differences in cultural norms, healthcare delivery and available resources.

Finally, implementation research should investigate effective strategies for integrating menopause education into routine primary healthcare services, workplace health programmes and community‐based initiatives. Such research could identify feasible and sustainable implementation strategies for improving menopause literacy and reducing inequities in access to menopause information and support.

## 5 Conclusion

This study provides a comprehensive understanding of the information needs and experiences of menopausal women by integrating the perspectives of both women and healthcare professionals. The findings demonstrate that women's informational needs extend beyond knowledge of menopausal symptoms and encompass understanding the menopausal transition, accessing personalised management strategies, navigating reliable sources of information and overcoming barriers embedded within healthcare systems and wider social environments.

The study further highlights that menopause literacy is a multidimensional concept shaped by interactions between individual, healthcare and societal factors. Addressing these interconnected dimensions requires coordinated efforts that combine anticipatory education, healthcare professional training, evidence‐based information resources, supportive social environments and accessible digital health solutions.

By providing an integrated understanding of menopause‐related information needs across individual, healthcare and societal levels, this study extends the evidence base supporting person‐centred menopause care. The findings may inform the development of educational programmes, digital health interventions and policies aimed at improving menopause literacy and supporting informed decision‐making in similar healthcare and socio‐cultural contexts.

## Author Contributions

**Najmeh Pourshahrokhi:** conceptualisation, investigation, writing – original draft, writing – review and editing, visualisation, validation, software, project administration, data curation, methodology. **Jamileh Farokhzadian:** conceptualisation, investigation, writing – review and editing, methodology, validation, visualisation, software, supervision, project administration. **Myra S. Hunter:** conceptualisation, writing – review and editing, project administration, supervision, formal analysis. **Saeedeh Azaraeen:** investigation, methodology, validation, visualisation, writing – review and editing, software, resources. **Leila Ahmadian:** conceptualisation, visualisation, validation, writing – review and editing, methodology, formal analysis, supervision, project administration.

## Funding

The authors have nothing to report.

## Ethics Statement

This study was approved by the Research Ethics Committee of Kerman University of Medical Sciences (Code: IR.KMU.REC.1403.087). In accordance with the committee's requirements, the research was conducted based on the principles of the Declaration of Helsinki and the guidelines of the Committee on Publication Ethics (COPE). To ensure ethical standards, participants were fully informed about the study's objectives and procedures, and all of them provided written informed consent prior to participation. Participation was voluntary, and participants were free to withdraw from the study at any time. No names were recorded or mentioned during the study, and the confidentiality of participant information was maintained throughout all stages of the research, from data collection to publication.

## Conflicts of Interest

The authors declare no conflicts of interest.

## Supporting information


Supporting File 1

## Data Availability

The datasets generated and analysed during the current study are not publicly available because they contain potentially identifiable qualitative interview data, and participants did not provide consent for public data sharing. De‐identified data may be made available from the corresponding author upon reasonable request and subject to approval by the Research Ethics Committee of Kerman University of Medical Sciences.
