# Supplementary material for: Menopause Literacy Gaps Across Individual, Healthcare and Societal Levels: A Qualitative Study of Women's Experiences and Healthcare Providers' Perspectives
Source: Health Expect. 2026 Oct 23;29(5):e70938. doi: 10.1111/hex.70938 (PMC13693505; doi:10.1111/hex.70938)
Supplement: Supplementary file 1 [file HEX-29-e70938-s001.docx]

| Symptoms | Parity | Marital status | Highest educational qualification | age | Participant |
| --- | --- | --- | --- | --- | --- |
| Anxiety, Forgetfulness, Depression and Hot flushes, Mood Swings, Insomnia | 2 | Widowed | University undergraduate | 49 | P1 |
| Brain Fog, Fatigue, Menstrual Migraines, Problems Concentrating, Dizziness, Skin Problems | 3 | Married | Diploma | 52 | P2 |
| Hot Flashes, Brain Fog, Fatigue, Urge to Urinate, Anger, Blurred Vision | 2 | Married | University postgraduate | 51 | P3 |
| Irregular Periods, Hot Flashes, Night Sweats Changes In Sexual Desire, Headaches, Vaginal Dryness | 2 | Married | University postgraduate | 48 | P4 |
| Mood Swings, Irregular Periods, Low Libido, Weight Gain | 1 | Married | University undergraduate | 46 | P5 |
| Night Sweats, Low Energy, Brain Fog, Trouble Sleeping | 0 | Single | University undergraduate | 49 | P6 |
| Changes to Periods, Brain Fog, Joint Pain, Insomnia, Fatigue | 2 | Married | University postgraduate | 53 | P7 |
| Weight Gain, Hot Flushes, Mood Swings, Insomnia, Anxiety | 1 | Married | University undergraduate | 46 | P8 |
| Hot Flashes, Brain Fog, Fatigue, Urge to Urinate, Anger, Blurred Vision | 1 | Widowed | University postgraduate | 56 | P9 |
| Changes in Sexual Function, Irregular Period, Mood Changes | 3 | Married | University undergraduate | 48 | P10 |
| Irregularities of Menstrual Period and Hot Flushes | 2 | Widowed | University undergraduate | 50 | P11 |
| Hot flashes, Night Sweats, Low Mood, Sweats, Heart Palpitations | 2 | Married | University postgraduate | 54 | P12 |
| Loss of Strength, Fatigue, Mood Changes, Heat Flushes, Increased Facial and Weight Gain | 0 | Single | University undergraduate | 51 | P13 |
| Mood Changes, Heat Flushes, Increased Facial | 1 | Widowed | Diploma | 45 | P14 |
| Irregular Periods, Hot Flashes, Night Sweats and Vaginal Dryness | 1 | Married | University undergraduate | 48 | P15 |
| Fatigue, Anxiety, Depression, Electric Shock Sensations | 0 | Single | University undergraduate | 49 | P16 |
| Urinary and Genital Problems, Fatigue and Hot Flashes | 3 | Married | University postgraduate | 47 | P17 |
| Urinary and Genital Problems, Restless Legs Syndrome, and Muscle Tension | 2 | Married | University undergraduate | 45 | P18 |
| Irregularities of menstrual period and Hot flushest | 4 | Married | Diploma | 47 | P19 |
| Fatigue, Anxiety, Hot flashes, Night Sweats and Vaginal dryness | 2 | Married | University undergraduate | 48 | P20 |
| Musculoskeletal Pain, Restless Legs Syndrome, Insomnia and Hot Flashes | 1 | Married | University postgraduate | 50 | P21 |
| Hot Flashes, Brain Fog, Fatigue, Anger, Blurred Vision | 0 | Single | University undergraduate | 48 | P22 |
| ***Work Experience (Year)*** | | ***Profession*** | | *Health professionals participant* | |
| 19 | | Nurse (PhD) | | P23 | |
| 29 | | Psychologist | | P24 | |
| 27 | | Gynecologist | | P25 | |
| 14 | | Gynecologist | | P26 | |
| 11 | | Social Medicine | | P27 | |
| 20 | | Psychologist | | P28 | |
| 15 | | Psychologist | | P29 | |
| 6 | | Mental Health Experts | | P30 | |
| 18 | | Midwifery Experts | | P31 | |
| 13 | | Midwifery Experts | | P32 | |
